# Supplementary material for: Downregulation of LOC441461 Promotes Cell Growth and Motility in Human Gastric Cancer
Source: Cancers (Basel). 2022 Feb 23;14(5):1149. doi: 10.3390/cancers14051149 (PMC8909665; doi:10.3390/cancers14051149)
Supplement: Supplementary file 1 [file cancers-14-01149-s001.zip › Supplementary materials legends_revised.pdf]

Figure S1. Knockdown of LOC441461 by transfection with si-LOC441461 or scrambled negative control (N.C) in SNU216 cells. The proliferation ability of N.C and si-LOC441461-transfected cells was examined using the CCK-8 so-lution at every 24 h. All experiments were performed in triplicate, and data are expressed as the mean ± standard deviation. **Figure S2.** MKN74 or SNU216 gastric cancer cells were seeded in Transwell plates to evaluate migration and invasion. (A) The results of migration and invasion assays with MKN74 and SNU216 cells stained with 0.01% crystal violet solution Scale bar = 200 μm. (B) Cell movement was quantified by eluting with 33% acetic acid and measuring absorbance at a wavelength of 590 nm using a plate reader. All experiments were performed in triplicate, and data were expressed as the mean ± standard deviation. **Figure S3.** Heatmap of the expression of the target genes of transcription factors dividing genes into LOC441461 knockdown and negative control groups. **Figure S4.** Heatmap of the expression of the target genes of transcription factors that did not divide genes into LOC441461 knockdown and negative control groups. **Figure S5.** IRF1 mRNA expression was upregulated in the LOC441461 knockdown group. qRT-PCR analysis of MKN74 cells transfected with scrambled negative control siRNA (N.C) or si-LOC441461. The data were normalized to GAPDH. All experiments were performed in triplicate and expressed as the mean ± standard deviation (***$p < 0.001$; Student's *t* test). **Table S1.** siRNA sequences and target RNA **Table S2.** Primer set used in qRT-PCR targeting LOC441461 including TRAIL, cyclin D1, IRF1, U6, and GAPDH. **Table S3.** The results of IG analysis using TCGA STAD RNA-seq data and TNM stage information from clinical data between stage I and IV. **Table S4.** Analysis of the expression of genes with IG >0 in TNM stages I and IV using t tests. **Table S5.** The results of log-rank test. **Table S6.** Differentially expressed genes between LOC441461 knockdown and N.C groups. KD is knockdown of LOC441461. N.C is negative controls**.** **Table S7.** Results of pathway enrichment analysis using the Reactome database and differentially expressed genes. Blue indicates a downregulated pathway in the LOC441461 knockdown group. Red indicates a upregulated pathway in the LOC441461 knockdown group. **Table S8.** Molecules that interact with LOC441461. **Table S9.** Significantly enriched transcription factors. **Table S10.** Target genes of each transcription factor.
