# Supplementary material for: Downregulation of LOC441461 Promotes Cell Growth and Motility in Human Gastric Cancer
Source: Cancers (Basel). 2022 Feb 23;14(5):1149. doi: 10.3390/cancers14051149 (PMC8909665; doi:10.3390/cancers14051149)
Supplement: Supplementary file 1 [file cancers-14-01149-s001.zip › Table S1 & S2.pdf]

Table S1. siRNA sequences and target RNA

siRNA	Sequence	Target RNA	Reference
si-LOC441461_318	5′-GGAACUGAAAUGUUGCCUUTT-3′,<br>5′-AAGGCAACAUUUCAGUUCCTT-3′	STX17-AS1	J.-H. Wang et al. Cancers (2020)
si-LOC441461_384	5′-GAUGGACUCACUAAAGGAUTT-3′,<br>5′-AUCCUUUAGUGAGUCCAUCTT-3′	STX17-AS1	J.-H. Wang et al. Cancers (2020)
si-LOC441461_432	5′-GCUGCUACAUUAACUGAUUTT-3′,<br>5′-AAUCAGUUAAUGUAGCAGCTT-3′	STX17-AS1	J.-H. Wang et al. Cancers (2020)

Table S2. Primer set used in qRT-PCR targeting LOC441461, TRAIL, Cyclin D1, IRF1, U6, and GAPDH.

Gene	Forward Primer	Reverse Primer
LOC441461	TGATAAGCTGTTTAAACCAGAACCG	GGCAACATTTCAGTTCCAGTG
TRAIL	TGGCAACTCCGTCAGCTCGTTA	AGCTGCTACTCTCTGAGGACCT
Cyclin D1	TCTACACCGACAACTCCATCCG	TCTGGCATTTTGGAGAGGAAGTG
IRF1	GAGGAGGTGAAAGACCAGAGCA	TAGCATCTCGGCTGGACTTCGA
U6	GGAACGATACAGAGAAGATTAGCA	GTGCAGGGTCCGAGGT
GAPDH	GTCTCCTCTGACTTCAACAGCG	ACCACCCTGTTGCTGTAGCCAA
