# Supplementary figures and images for: Downregulation of LOC441461 Promotes Cell Growth and Motility in Human Gastric Cancer
Source: Cancers (Basel). 2022 Feb 23;14(5):1149. doi: 10.3390/cancers14051149 (PMC8909665; doi:10.3390/cancers14051149)

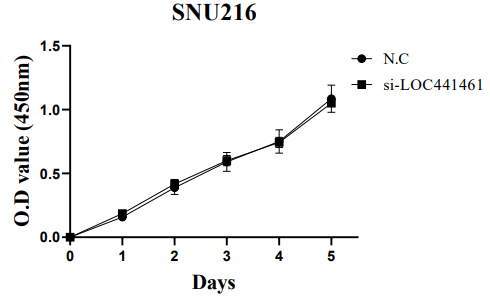

Supplement: Supplementary file 1 [file cancers-14-01149-s001.zip › Figure S1.png]

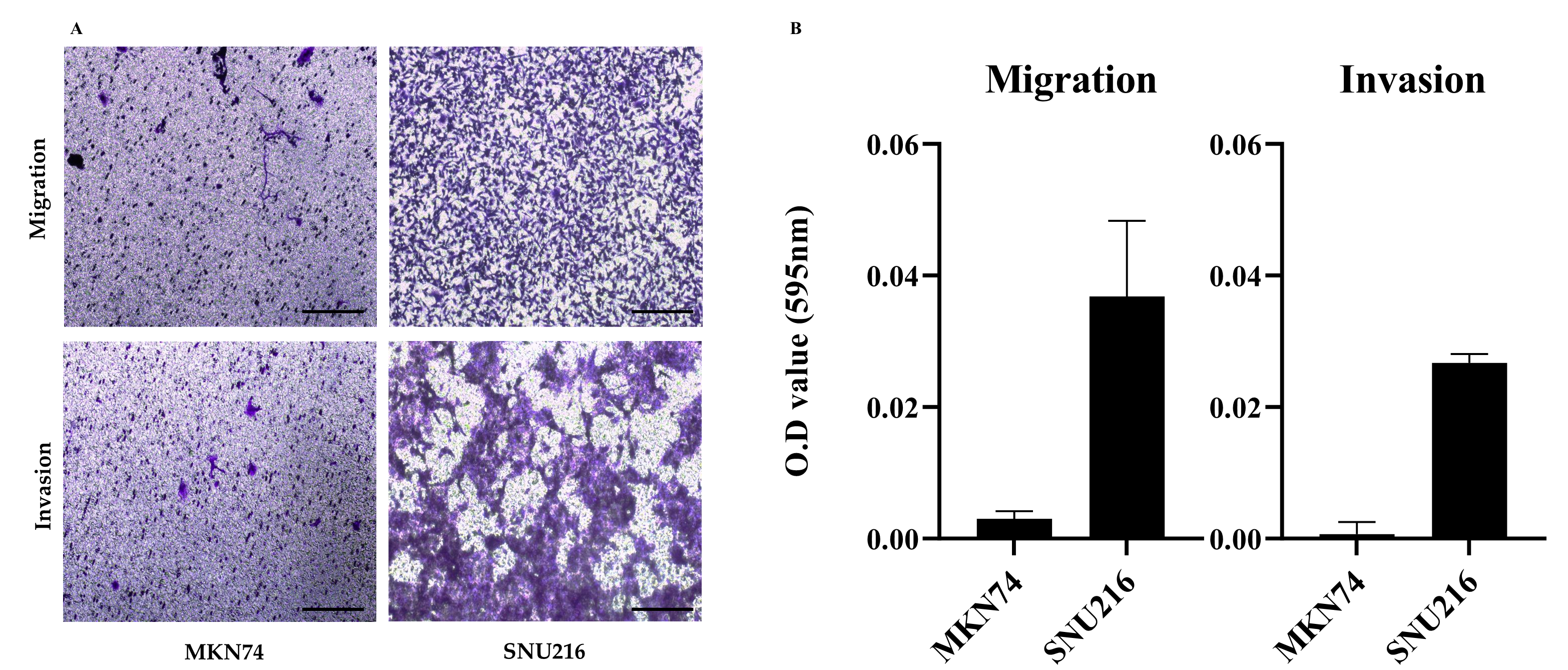

Supplement: Supplementary file 1 [file cancers-14-01149-s001.zip › Figure S2.png]

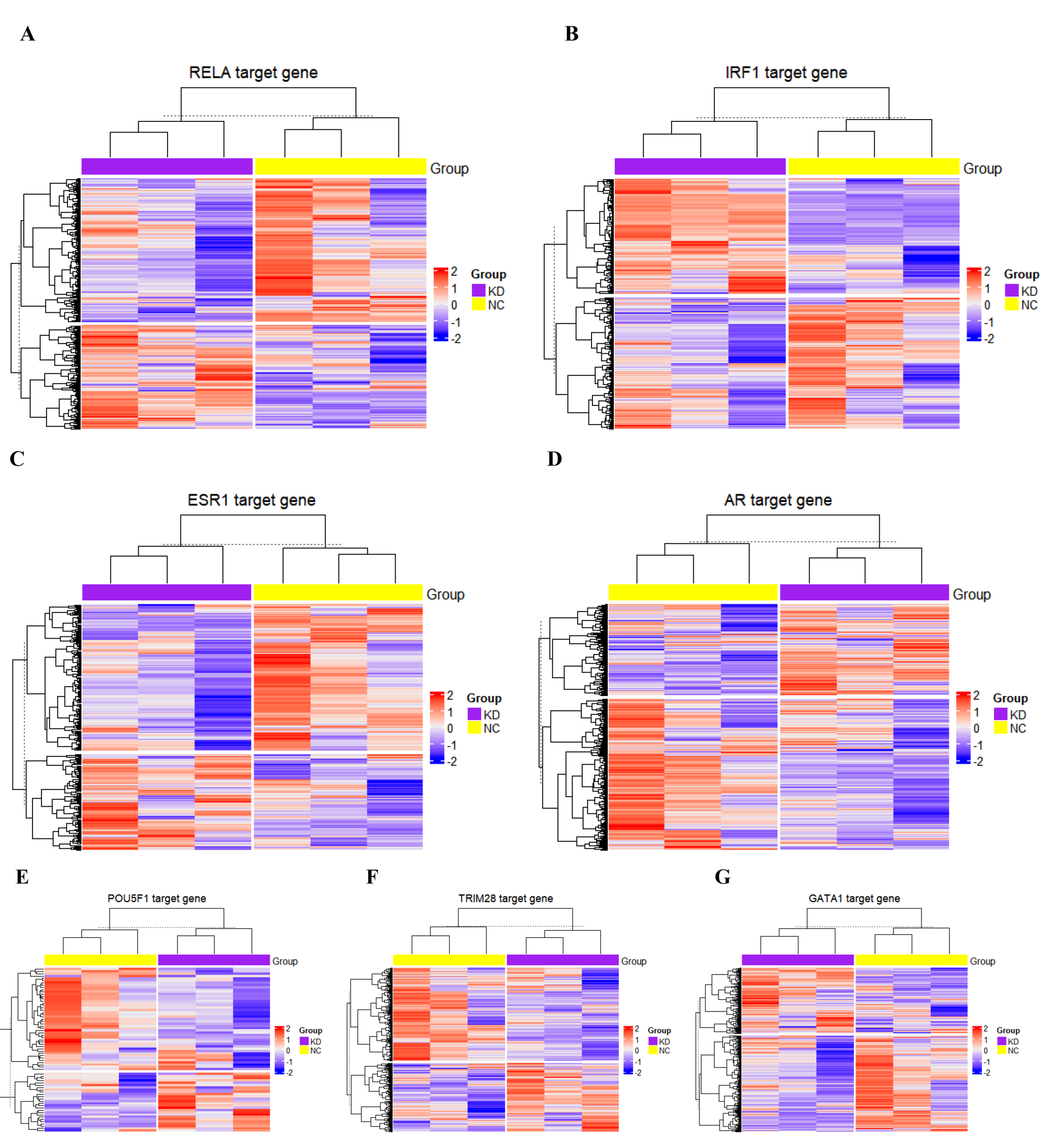

Supplement: Supplementary file 1 [file cancers-14-01149-s001.zip › Figure S3.png]

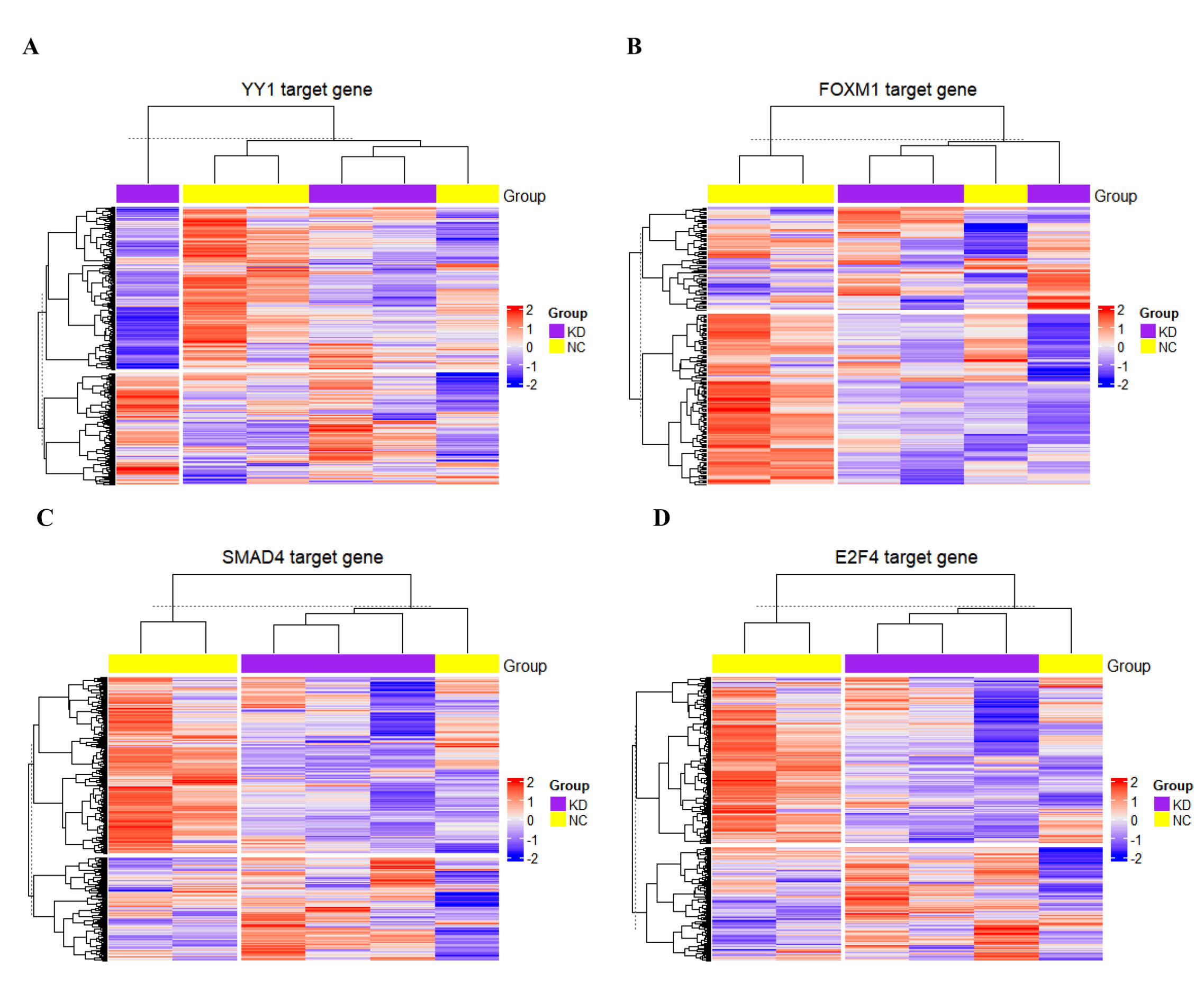

Supplement: Supplementary file 1 [file cancers-14-01149-s001.zip › Figure S4.png]

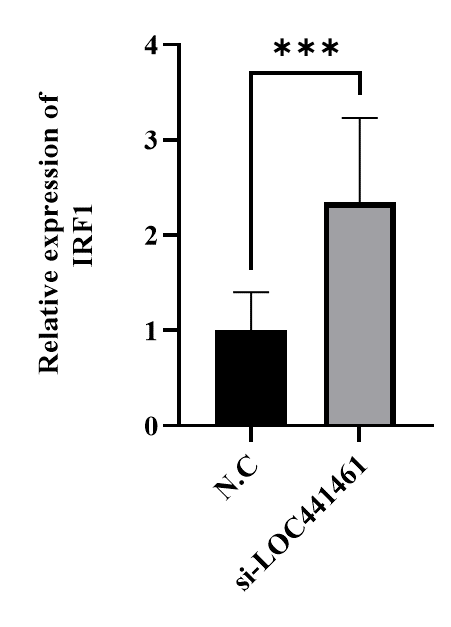

Supplement: Supplementary file 1 [file cancers-14-01149-s001.zip › Figure S5.png]
